# Immunosuppressants in dermatology on vaccine immunogenicity: a prospective cohort study of pemphigus patients in the pandemic

**DOI:** 10.3389/fimmu.2024.1506962

**Published:** 2024-11-22

**Authors:** Kun-Lin Lu, Hua-En Lee, Chun-Bing Chen, Rosaline Chung-Yee Hui, Ya-Ching Chang, Chun-Wei Lu, Chuang-Wei Wang, Wen-Hung Chung

**Affiliations:** ^1^ Department of Dermatology, Drug Hypersensitivity Clinical and Research Center, Chang Gung Memorial Hospital, Taipei, Keelung, Taiwan; ^2^ College of Medicine, Chang Gung University, Taoyuan, Taiwan; ^3^ Chang Gung Immunology Consortium, Chang Gung Memorial Hospital and Chang Gung University, Tao-Yuan, Taiwan; ^4^ Department of Dermatology, Xiamen Chang Gung Hospital, Xiamen, China; ^5^ Keelung Chang Gung Memorial Hospital, Keelung, Taiwan; ^6^ Cancer Vaccine and Immune Cell Therapy Core Laboratory, Department of Medical Research, Chang Gung Memorial Hospital, Linkou, Taiwan; ^7^ Immune-Oncology Center of Excellence, Chang Gung Memorial Hospital, Linkou, Taiwan; ^8^ Graduate Institute of Clinical Medical Sciences, College of Medicine, Chang Gung University, Taoyuan, Taiwan; ^9^ Whole-Genome Research Core Laboratory of Human Diseases, Chang Gung Memorial Hospital, Keelung, Taiwan; ^10^ School of Medicine, National Tsing Hua University, Hsinchu, Taiwan; ^11^ Allergology Consortium, Xiamen Chang Gung Hospital, Xiamen, China; ^12^ Department of Dermatology, Beijing Tsinghua Chang Gung Hospital, School of Clinical Medicine, Tsinghua University, Beijing, China; ^13^ Department of Dermatology, Ruijin Hospital, School of Medicine, Shanghai Jiao Tong University, Shanghai, China; ^14^ Genomic Medicine Core Laboratory, Chang Gung Memorial Hospital, Linkou, Taiwan

**Keywords:** vaccine immunogenicity, anti-viral humoral immunity, anti-viral t-cell response, rituximab, azathioprine, pemphigus vulgaris

## Abstract

**Introduction:**

Both cellular and humoral responses are important for vaccine protection, but recommendations on immunosuppressants in dermatology are largely based on pre-pandemic experiences. This study aimed to investigate the impacts of immunosuppressants on humoral and cellular immunogenicity to COVID-19 vaccinations in pemphigus patients.

**Methods:**

SARS-CoV-2-naïve pemphigus patients and age-, and sex-matched healthy controls were recruited from multiple tertiary medical centers during 2021-2023. Anti-spike protein-related T-cell responses, antibody titers, and high-parameter cell analysis of the peripheral blood were utilized to investigate the inhibitory effects of immunosuppressants, including rituximab and azathioprine.

**Results:**

A total of 32 patients and 120 healthy controls were enrolled. COVID-19 vaccinations spaced at least six months after the last rituximab infusion did not cause a significant difference in anti-viral T-cell or antibody responses between rituximab-naïve and rituximab-treated patients. All pemphigus patients demonstrated improved antibody responses after the third vaccination and none of them suffered from severe COVID-19 illness. Intriguingly, we found that daily dosages of 100 mg or more of azathioprine were linked to significantly decreased anti-viral T-cell responses induced by the vaccination (mean of fold change [SD]; higher azathioprine dosage = 0.70 [0.61] folds vs. lower azathioprine dosage = 2.11 [1.03] folds; *p* = 0.044).

**Conclusion:**

Except for a subset of patients with unrecovered B-cell deficiency, rituximab infusion with proper scheduling of vaccination preserved better anti-viral T-cell responses and did not lead to hindered antibody responses in pemphigus patients. All pemphigus patients benefited from receiving the third booster regardless of B-cell status.

## 1 Introduction

Vaccination has long been one of the most effective public health interventions and has become increasingly relevant as the global pandemic of coronavirus disease 2019 (COVID-19) evolves. Pemphigus vulgaris and pemphigus foliaceus, autoimmune bullous diseases caused by autoantibodies targeting desmogleins within the skin, generally require systemic corticosteroids, traditional immunosuppressants, or the combination of them for long-term maintenance treatments. B-cell depletion therapy with anti-CD20 agents such as rituximab has been proven as a first-line treatment option for moderate-to-severe pemphigus (1). However, these immunosuppressants are known to suppress immune cells and hamper vaccine responses (2, 3), while the detrimental effects of rituximab are further long-lasting on vaccine-related antibody responses (4). For instance, previous studies had reported that receiving methotrexate, tumor necrosis factor inhibitor, or Janus kinase inhibitor is linked to poorer humoral and cellular immune responses to the COVID-19 vaccines (3, 5, 6), even after receiving their third vaccination (7), while recent rituximab infusion is related to poor antiviral antibody responses (8–10). Due to the high varieties of immunosuppressants taken by the patients recruited and also the heterogenicity in the disease natures included, previous cohorts often face many confounding factors before delineating the detrimental effects brought by each specific medication (11).

To date, little is known about which treatment combined with what kind of vaccination schedule would benefit the patients with pemphigus the most. Therefore, our study aims to evaluate the effects of rituximab and immunosuppressants (including azathioprine and corticosteroids) on both humoral and cellular anti-viral responses to COVID-19 vaccinations in patients with pemphigus. We also investigated their associations with peripheral blood cellular subpopulations after vaccination and aimed to generate a preferable strategy integrating COVID-19 vaccination and treatment plans for patients with pemphigus, which may also be of reference value for other autoimmune diseases.

## 2 Materials and methods

### 2.1 Study design and population

Our prospective pemphigus cohort was established at the start of 2021 at Chang Gung Memorial Hospital, Linkou, Taipei, and Keelung branch. Under informed consent, we recruited adult patients with a biopsy-proven diagnosis of pemphigus vulgaris, pemphigus foliaceus, or paraneoplastic pemphigus and followed up with them regularly every two to four weeks through their second and/or third dose of COVID-19 vaccination until the end of 2023. During the longitudinal follow-up, we planned to draw their post-vaccination blood sampling 1-2 months after vaccination. Exclusion criteria include COVID-19 infection before and during the follow-up period (i.e., having any positive reverse transcriptase–polymerase chain reaction result by nose swab, or being reported by the National Infectious Disease Reporting System before and during the follow-up period), loss to follow up, and patients identified with other immunosuppressed condition. We also recruited age-, and sex-matched healthy controls as the reference group, and collected their post-vaccinated peripheral blood for comparison. Each subject enrolled in this study provided written informed consent to the publication of their case details, and the study protocol was approved by the institutional review board (IRB) and ethics committee of each hospital based on Taiwan’s laws and regulations (IRB No. 2206120006 and IRB No. 202101436B0).

### 2.2 Anti-viral antibody responses

Peripheral blood mononuclear cells (PBMCs) were isolated from patients’ whole blood samples using Ficoll-Paque (Pharmacia Fine Chemicals, Uppsala, Sweden) density gradient centrifugation, and were further tested for anti-viral T-cell responses by interferon (IFN)-γ-releasing test and lymphocytic subpopulations by flow cytometry. The IgG antibodies targeting the spike protein of severe acute respiratory syndrome coronavirus 2 (SARS-CoV-2) within the plasma samples were measured by enzyme-linked immunosorbent assay (ELISA) supplied by Beijing Wantai Biological Pharmacy Enterprise Co., Ltd., according to the manufacturer’s instructions. In brief, double-antigen sandwich ELISA was developed using mammalian cell-expressed recombination antigens containing the receptor-binding domain (RBD) of the spike protein of SARS-CoV-2 (100 ng) as the immobilized and horseradish peroxidase-conjugated antigens. IgG antibodies (ab6759, Abcam; Waltham, USA) and IgE antibodies (ab99806, Abcam) were detected using an indirect ELISA kit. The specificity of the assays for IgG was determined to be 99.0% (195/197) by testing samples collected from healthy individuals before the outbreak of SARS-CoV-2.

### 2.3 Anti-viral T-cell responses

To examine anti-viral T-cell responses, 1.0 × 10^6^ PBMCs were cultured in 96-well microplates in RPMI-1640 medium (GIBCO Invitrogen, Life Technologies, Carlsbad, CA) supplemented with 10% human AB serum (Sigma-Aldrich, Darmstadt, Germany), IL-7 (Invitrogen), and COVID-19 spike protein and being tested at 37°C in 5% CO_2_ for 1 week. In addition, the phosphate-buffered saline was added to the medium as the solvent control, and phytohemagglutinin at a concentration of 10 μg/ml was used as the positive control. After culturing for 1 day, the culture supernatants were collected to measure the secretions of IFN-γ, which is regarded as the T-cell activation maker, by DuoSet ELISA kit (Cat. #DY285B; R&D Systems, Minneapolis, MN, USA).

### 2.4 High-parameter cell analysis

Flow cytometry was carried out using distinct fluorochrome-conjugated monoclonal antibodies that recognize a variety of lymphocytic markers, including CD3, CD4, CD8, CD19, CD21, CD24, CD27, CD28, CD38, CD57, CD45RA, CCR7, PD1, IgM, and IgD. These monoclonal antibodies were labeled with selected lasers from various wavelengths with multiple power ratings. The cells were examined utilizing flow cytometry on the BD Symphony LSR II cell analyzer (BD Biosciences), and data was analyzed with the BD Paint-A-Gate Pro™ Software and FlowJo™ Software.

### 2.5 Statistical analysis

The anti-viral antibody titers were analyzed after log_10_-transformation. The fold-changes of IFN-γ levels were calculated by dividing the data by solvent controls. Continuous variables were first examined for each of their distribution by the Shapiro–Wilk test and the F-test to identify unequal variances. Non-normally distributed variants underwent log transformation to conform to normality. Normally distributed quantitative variables were summarized as means and standard deviations (SDs), while quantitative variables were reported as median and interquartile range (IQR), whereas categorical variables were denoted as absolute count and percentage. The differences in the continuous variables between the groups were analyzed by the two-sample *t*-test, unpaired *t*-test with Welch’s correction, or Mann–Whitney U test as appropriate. The correlations between two categorical variables were analyzed by the Chi-square test or Fisher exact test, while those between continuous or ordinal variables were analyzed by Pearson’s correlation coefficient or Spearman’s rank-order correlation. Missing data was prevented before gathering the data by excluding patients without blood survey results, and deletion was done before analysis. All *P*-values were two-tailed, and a *P*-value of < 0.05 was considered statistically significant. Statistical analyses were performed using the SPSS software Version 18.0 (SPSS Inc, Chicago, IL). Graphs were created by utilizing GraphPad Prism software version 9.0 (GraphPad Software).

## 3 Results

### 3.1 Cohort characteristic

Patient characteristics are demonstrated in **Table 1**. In brief, our cohort included 32 eligible patients, 16 patients completed their second vaccination, 10 patients completed their third vaccination, and 6 of them completed both their second and third vaccination during the follow-up period (before the end of 2023), with pemphigus vulgaris as the major underlying condition (15 [68.2%] and 16 [100%] in patients receiving their second and third dose of vaccination, respectively). The patients randomly received either an mRNA-based vaccine (i.e., mRNA-1273 or BNT162b2 vaccination) or a non-mRNA-based vaccine (such as the ChAdOx1-S vaccination) as their second or third COVID-19 vaccination. During the follow-up period, all patients were under stable conditions and low-dose methylprednisolone, azathioprine, or both for long-term maintenance therapy, with no episode of acute flare-up. A history of receiving rituximab was documented in 13 [59.1%] patients before receiving their second vaccination, and 7 [43.8%] patients before receiving their third vaccination. To minimize the inhibitory effect of rituximab on antibody responses, we managed to schedule the vaccinations at least six months after the last infusion. The median interval between the vaccination and post-vaccination blood draw was 6.7 [IQR, 3.4-11.3] weeks. Of note, we still identified 7 [31.8%] and 3 [18.8%] patients with B-cell deficiency (defined as peripheral B-cell count <30 cell/μL) at the time when they received their second and third vaccination, respectively. Adequate post-vaccination anti-viral antibody responses were found in 11 [50%] and 14 [87.5%] patients, while adequate T-cell responses (defined as ≧1.8-fold change) were identified in 6 [40%] and 3 [20%] patients receiving their second and third vaccination, respectively. None of the patients developed severe COVID-19 infection during a minimum 1-year follow-up period after vaccination.

**Table 1:** Patient characteristics.

Characteristics	2nd vaccination	3rd vaccination
Age, year, mean (SD)	54.0 (12.4)	51.5 (13.7)
Sex, no. (%)		
Male	11 (50)	3 (18.8)
Female	11 (50)	13 (81.2)
Diagnosis, no (%)		
Pemphigus vulgaris	15 (68)	16 (100)
Pemphigus foliaceus	6 (30)	0 (0)
Paraneoplastic pemphigus	1 (5)	0 (0)
Vaccination received, no. (%)		
mRNA-based vaccine (mRNA-1273 or BNT162b2)	17 (77.3)	14 (87.5)
Non-mRNA-based vaccine (ChAdOx1-S)	5 (22.7)	2 (12.5)
Methylprednisolone dosage, median (IQR), mg/day	1.6 (0-5.5)	1.2 (0-5.0)
Azathioprine dosage, median (IQR), mg/day	50 (0-75)	15 (0-50)
Previous rituximab use, no. (%)	13 (59.1)	7 (43.8)
Peripheral B-cell percentage, % (SD)	8.1 (8.4)	8.6 (7.8)
Peripheral B-cell count, cell/μL (SD)	136.9 (156.5)	333.9 (550.1)
Peripheral B-cell deficiency, no. (%)	7 (31.8)	3 (18.8)
Antibody responses, median (IQR), AU/ml	1401.6 (197.8-2543.9)	6403.5 (2424.2-11306.2)
Adequate antibody response, no. (%)	11 (50)	14 (87.5)
T-cell responses, fold change (SD)	1.82 (1.10)	1.1 (1.4)
Adequate T-cell response, no. (%)	6 (40)	3 (20)

### 3.2 Humoral immune responses

**Figure 1:**
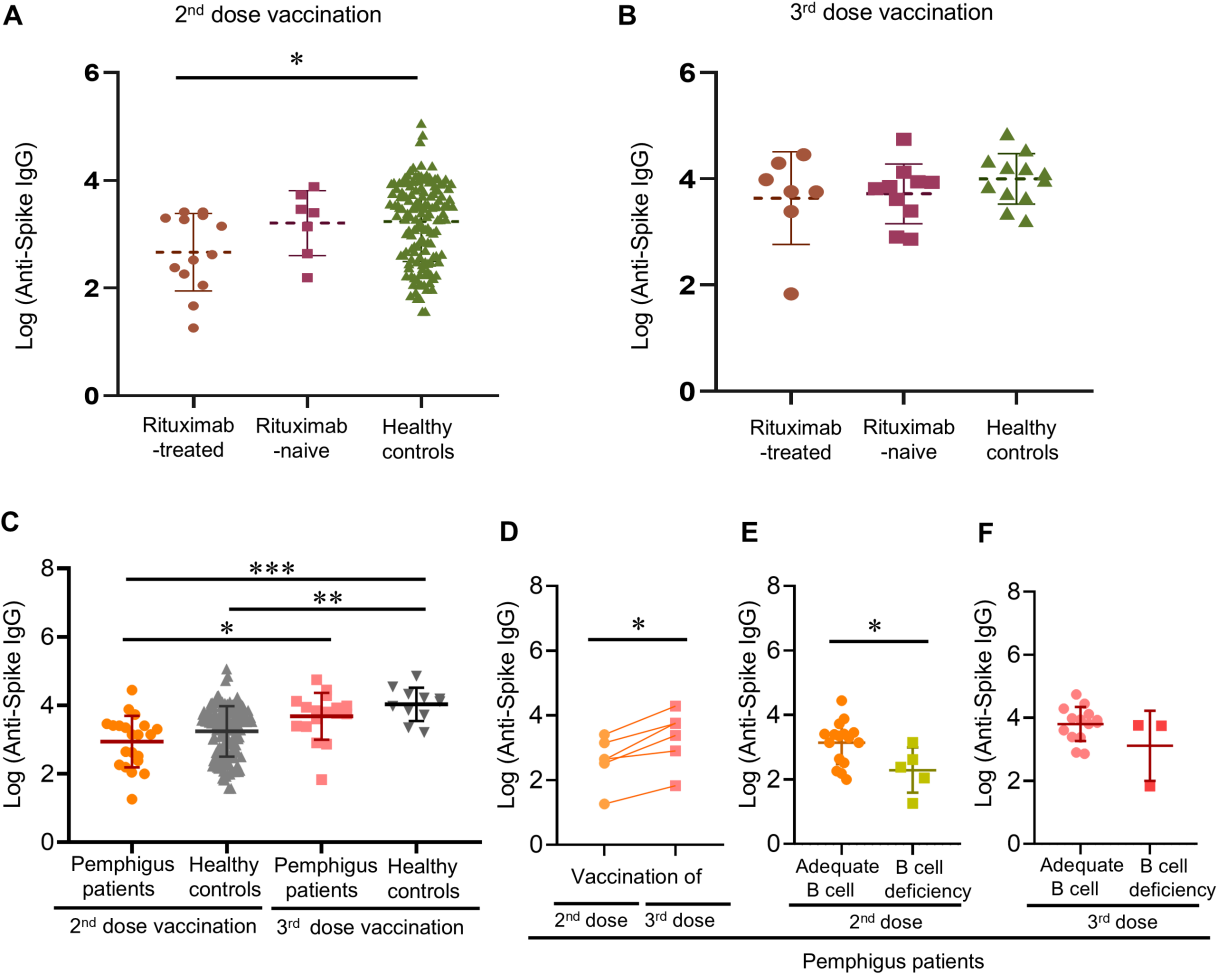
Post-vaccinated anti-viral antibody responses of pemphigus patients or healthy controls. **(A)** Post-vaccinated anti-viral antibody responses after the second vaccination, or **(B)** the third vaccination; **(C)** The differences in anti-viral antibody titers after receiving the second or third vaccination between the patients and the healthy controls; **(D)** Longitudinal follow-up of post-vaccination anti-viral antibody responses to the second and the third vaccination of pemphigus patients; **(E)** The influence of peripheral B-cell deficiency on post-vaccination anti-viral antibody responses after the second dose, and **(F)** the third dose. Data are presented as the mean ± SEM. *p < 0.05, **p < 0.01, ***p < 0.001, calculated by 2-tailed two-sample t-test, unpaired t-test with Welch’s correction, or paired sample t-test as appropriate.

The medians of anti-viral antibody concentrations of pemphigus patients were 1401.6 [IQR, 197.8-2543.9] AU/mL of the second vaccination, and 6403.5 [IQR, 2424.2-11306.2] AU/mL of the third vaccination. There were no differences in the time intervals between rituximab infusion and the second or third vaccination, while the numerical differences in antibody concentrations between patients with and without a history of rituximab infusion did not reach statistical significance (rituximab-naïve patients = 2537.8 [IQR, 441.1-5407.3] vs. rituximab-treated patients = 419.9 [IQR, 147.5-2108.0] AU/mL in the second vaccination group; rituximab-naïve patients = 6701.7 [IQR, 2050.5-9847.0] vs. rituximab-treated patients = 5800.4 [IQR, 2379.0-19294.5] AU/mL in the third vaccination group) (as shown in **Table 2**). Using age- and sex-matched healthy controls as references (120 participants; mean [SD] age, 45.8 [13.1] years; 45 [37.5%] men), patients infused with rituximab before were found to have significantly inferior antibody responses to their second dose of COVID-19 vaccination as compared to healthy controls (rituximab group = 419.9 [IQR, 147.5-2108] vs. healthy controls = 2537 [IQR, 411-6657] AU/mL; *p* = 0.016) (**Figure 1A**). However, this difference was no longer found in the third vaccination group (rituximab group = 5800 [IQR, 2379-19300] vs. healthy controls = 11040 [IQR, 4608-20000] AU/mL; *p* = 0.24), demonstrating improved antibody responses comparable to healthy controls (**Figure 1B**). Additionally, significantly better antibody responses were found in patients and healthy controls that received their third vaccination, as compared to patients or healthy controls that only received their second dose (**Figure 1C**). Six patients received both their second and third vaccination during the longitudinal follow-up, and all of them demonstrated improved antibody responses after their third vaccination despite three of them still having peripheral B-cell deficiency (5526 [IQR, 794.0-5800] vs. 420 [IQR, 239.0-1410] AU/mL, *p* = 0.0023) (**Figure 1D**). Intriguingly, poor antibody responses to the second vaccination were related to B-cell deficiency (239.0 [IQR, 47.00-1410] vs. 2108 [IQR, 358.0-2556] AU/mL; *p* = 0.023) (**Figure 1E**), but this correlation was no longer evident after the patients received their third vaccination (**Figure 1F**).

**Table 2:** Subgroup analysis divided by a history of rituximab infusion.

	2nd vaccination		*P*-value	3rd vaccination		*P*-value
History of rituximab	Yes, N=13	No, N=9		Yes, N=7	No, N=9	
Methylprednisolone dosage, median (IQR), mg/day	1.1(0-4.0)	4.0(0-8.0)	0.428	4.0(0-6.0)	0.56(0-3.0)	0.417
Azathioprine dosage, median (IQR), mg/day	0(0-50)	75(50-100)	0.016	29(0-50)	7.0(0-50)	0.669
Time since last rituximab, median (IQR), days	443.0 (243.5-673.5)	N.A.	N.A.	381.0 (274.0-1448.0)	N.A.	N.A.
Total dosage of rituximab, mg (SD)	2260 (1230)	N.A.	N.A.	2271 (1034)	N.A.	N.A.
Peripheral B-cell percentage, median (IQR), %	3.20 (0.25-9.50)	13.9 (4.7-19.0)	0.126	14.1 (0.10-17.90)	7.20 (3.65-15.6)	0.299
Peripheral B-cell count, median (IQR), cell	24.0 (4.0-196.5)	217.0 (65.0-362.5)	0.084	300(5.0-507.0)	90.0 (59.5-420.5)	0.758
Peripheral B-cell deficiency, no. (%)	7 (53.8)	0 (0)	0.036	3 (42.8)	0 (0)	0.029
Antibody responses, median (IQR), AU/ml	419.9 (147.5-2108.0)	2537.8 (441.1-5407.3)	0.088	5800.4 (2379.0-19294.5)	6701.7 (2050.5-9847.0)	0.962
T-cell responses, fold change (SD)	2.69 (3.06)	0.52 (0.61)	0.189	1.16 (1.18)	1.02 (1.54)	0.85
Adequate T-cell response, no. (%)	6 (46.2)	0 (0)	0.057	1 (14.3)	2 (22.2)	0.792

### 3.3 Cellular immune responses

**Figure 2:**
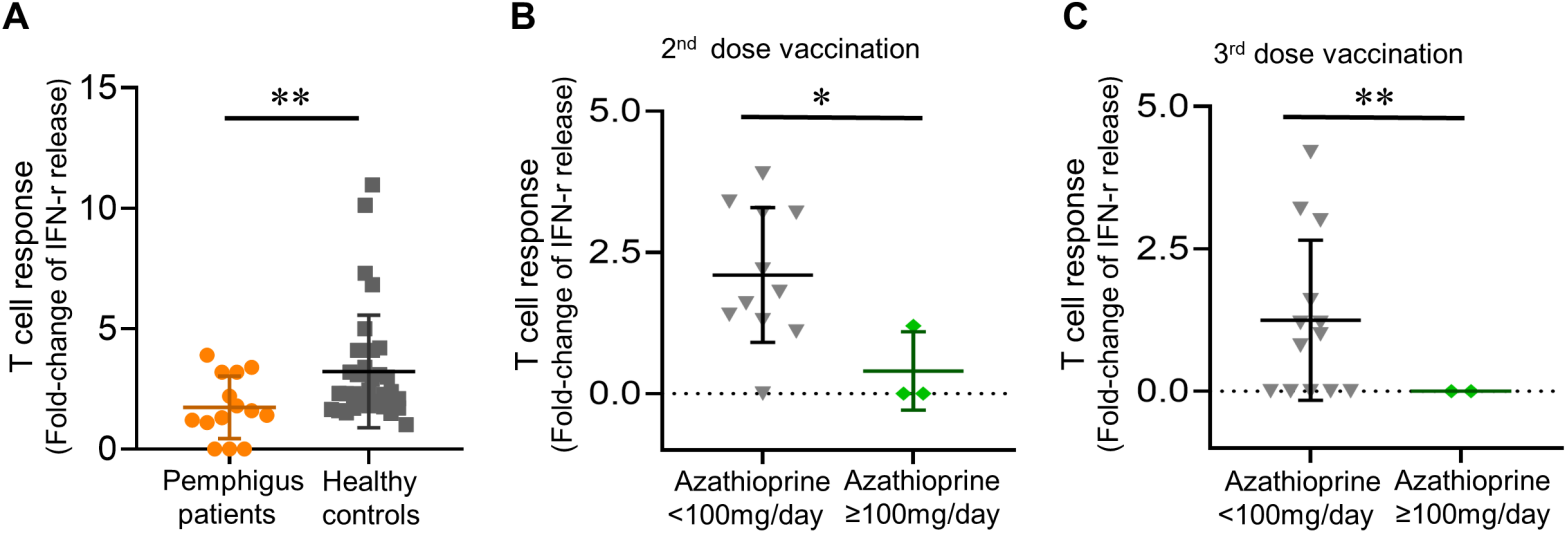
Post-vaccinated anti-viral T cell responses. **(A)** Post-vaccinated anti-viral T-cell responses in patients and healthy controls; **(B)** Daily azathioprine dosage not less than 100mg/day linked to a trend of diminished anti-viral T-cell responses, both in patients receiving their second dose, and **(C)** in patients receiving their third dose of vaccination. Data are presented as the mean ± SEM. *p < 0.05, **p < 0.01, calculated by 2-tailed two-sample *t*-test, unpaired *t*-test with Welch’s correction, or Mann–Whitney U test as appropriate.

Of the 14 patients tested among the second, and the 15 patients examined among the third vaccination cohort, the mean (SD) anti-viral T-cell responses were 1.82 (1.10)-fold after the second vaccination, and 1.10 (1.40)-fold after the third vaccination. Healthy controls demonstrated significantly better anti-viral T-cell responses as compared to pemphigus patients after receiving their second vaccination (mean [SD]-fold, 1.74 [1.30] vs. 3.22 [2.34]; *p* = 0.0077) (**Figure 2A**).

Intriguingly, we found that taking oral azathioprine not less than 100 mg per day was linked to significantly diminished anti-viral T-cell responses as compared to other patients in the pemphigus cohort (mean [SD] fold change, 0.40 [0.69] vs. 2.10 [1.19]; *p* = 0.020 in the second vaccination cohort, and 0 [0] vs. 1.25 [1.41]; *p* = 0.008 in the third vaccination cohort) (**Figure 2B, C**). On the other hand, methylprednisolone dosage did not correlate with altered T-cell responses (data not shown).

### 3.4 The impacts of rituximab

As aforementioned, we postponed vaccination after each rituximab infusion. The median interval between the last rituximab infusion and the second COVID-19 vaccination was 443.0 [IQR, 243.5-673.5] days, while the median interval for the third vaccination was 381.0 [IQR, 274.0-1448.0] days. After dividing patients into those with a history of rituximab treatment and the others that remained rituximab-naïve for comparison, all patients identified with B-cell deficiencies were in the rituximab-treated group (**Table 2**). However, a history of receiving rituximab was not directly associated with altered antibody responses within patients with pemphigus despite the increased risk for B-cell deficiency (**Figure 1A**).

We found that the maintenance therapies for pemphigus in the rituximab-naïve group depended more on azathioprine (but not methylprednisolone) as compared to the rituximab-treated group (mean [SD] mg/day, 78.5 [22.5] vs. 27.0 [39.0], *p* = 0.0048). This finding coincided with a significantly decreased anti-viral T-cell responses in the rituximab-naïve group (mean [SD]-fold, 0.70 [0.84] vs. 2.70 [3.06]; *p* = 0.032).

As expected, the time interval between vaccination and the last rituximab infusion positively correlated to peripheral B-cell counts in patients receiving their second vaccination (*p* = 0.046), but it did not directly associate with antibody responses (data not shown). Of note, peripheral B-cell count did correlate to anti-viral antibody responses in our cohort, especially in patients receiving their second vaccination (Spearman’s rho [95% CI], 0.58 [0.02-0.86]; *p* = 0.041). Neither B-cell deficiency nor poor antibody responses associated with the cumulative total dosage of rituximab received (data not shown).

### 3.5 Subpopulations of peripheral lymphocytes

**Figure 3:**
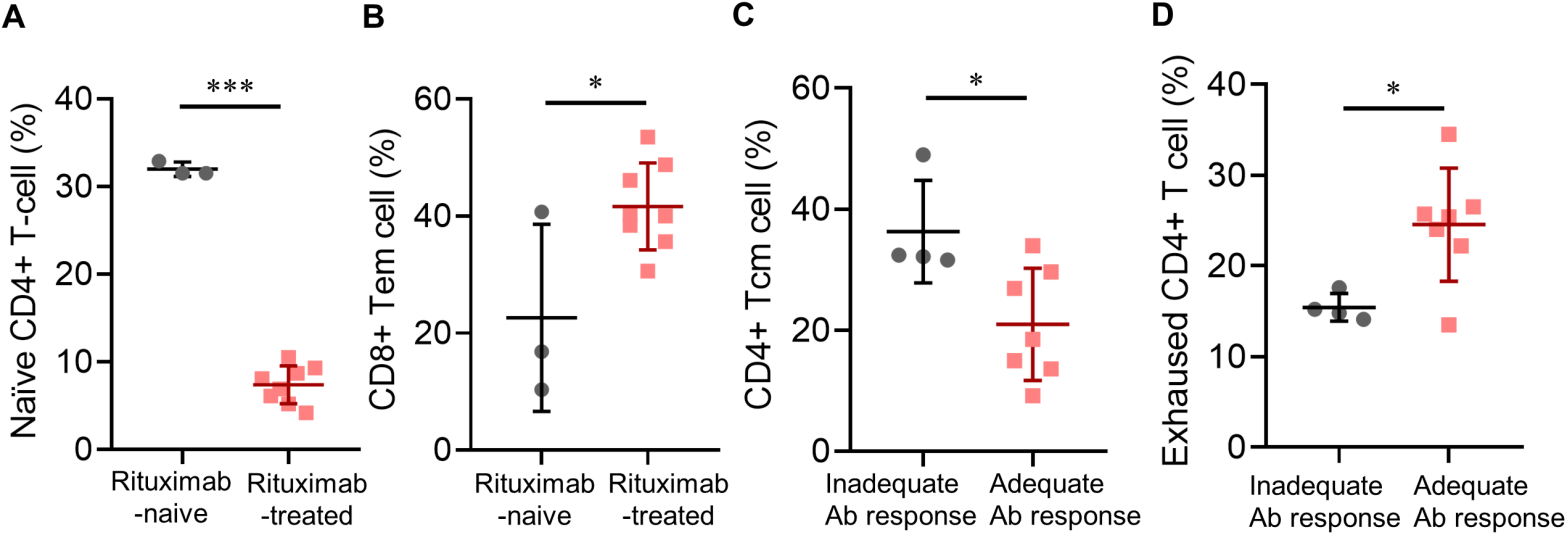
Post-vaccinated subpopulations of immune cells in pemphigus patients. **(A)** Associations of a history of rituximab treatment with post-vaccinated naïve CD4^+^ T-cell percentage, or **(B)** CD8^+^ effector memory T-cell percentage; **(C)** Associations of anti-viral antibody responses with post-vaccinated CD4^+^ central memory T-cell percentage, or **(D)** exhausted CD4^+^ T-cell percentage. Data are presented as the mean ± SEM. *p < 0.05, ***p < 0.001, calculated by 2-tailed two-sample *t*-test, unpaired *t*-test with Welch’s correction, or Mann–Whitney U test as appropriate.

By utilizing high-parameter cell analysis, we analyzed the differences in cellular compositions of each T- or B-cell subpopulation (**Supplementary Figures S1**, **S2**). In patients with a history of rituximab use, there were significantly lower frequencies of multiple B-cell compartments, including unswitched memory B cells, marginal B cells, and CD21^low^ B cells, even after adjusting for peripheral B cell counts, whereas decreases in the reservoir of naïve B cells were more prominent in patients failing B-cell repopulation. Intriguingly, a history of rituximab use was also significantly associated with decreased percentages of naïve CD4^+^ T cells (mean [SD] %, 7.4 [2.2] vs. 32.0 [0.8]; *p* < 0.0001) (**Figure 3A**), but increased CD8^+^ effector memory T-cell counts (mean [SD] %, 41.6 [7.4] vs. 22.6 [16.0]; *p* = 0.020) (**Figure 3B**). These trends were also noticed in patients receiving their third vaccination, though some did not reach statistical significance.

Looking into the associations between post-vaccination subpopulations of lymphocytes and antibody responses, we found that patients with adequate antibody responses had significantly lower percentages of CD4^+^ central memory T cells (mean [SD] cells, 21.0 [9.3] vs. 36.3 [8.5]; *p* = 0.023) (**Figure 3C**), significantly higher percentages of exhausted CD4^+^ T cells (defined as CD3^+^CD4^+^CD57^-^PD-1^high^ T cells, mean [SD] %, 24.5 [6.2] vs. 15.4 [1.5]; *p* = 0.020) (**Figure 3D**), and near-significant elevations in percentages of both CD8^+^ T cells and naïve CD4^+^ T cells.

## 4 Discussion

The COVID-19 pandemic is reminiscent of the dilemma in our daily practice, which is to maintain adequate immunosuppression for controlling autoimmune diseases while risking a higher probability of non-responsiveness to vaccination (12, 13). B cell-depletion therapies are known for their low seroconversion rate (14–16), while other treatments such as corticosteroids, methotrexate, and azathioprine also pose risks to poor immunogenicity (17, 18).

Previous studies have suggested that the shorter the time interval between vaccination and the last anti-CD20 treatment, the poorer the antibody responses would be (19–22), and recommendations on the proper time interval range grossly from three to six months (23, 24). However, there were still multiple reports of large proportions of non-responders even with six-month rituximab-to-vaccination intervals (25, 26). As for pemphigus, anti-CD20 treatment induces a reset of the B-cell repertoire and demonstrates its long-term disease control following standard protocols (27). This not only facilitates studying its immunomodulatory effects in a patient population that is less confounded by other concurrent immunomodulation or hematologic disorders but also unfolds the opportunities for exploring optimal time windows for successful vaccination.

Our prospective cohort study showed that pemphigus patients with a history of rituximab infusion, even spaced apart from vaccination for at least six months, were still at higher risk for B-cell deficiency, while a following booster could improve anti-viral humoral immunity with comparable anti-viral T-cell responses, overall in line with previous findings (28, 29). Of note, rituximab-naïve patients tend to require a higher dosage of azathioprine for long-term disease control and were linked to poorer anti-viral T-cell responses brought by vaccination, especially in patients receiving not less than 100 mg of azathioprine per day. In contrast to current recommendations on COVID-19 vaccination for dermatological patients that consider azathioprine as having little to no significant impairment on vaccine immunogenicity (12, 30), our findings suggest a need to withhold azathioprine before vaccination. Our results also suggested that proper rituximab scheduling could diminish its inhibitory effect on antibody responses while showing non-inferior anti-viral T-cell responses to vaccination, echoing the results of previous reports (31, 32). The possible mechanisms for rituximab to even enhance T-cell responses may involve compromised regulatory B cells (33), the absence of antigen clearance by vaccine-induced antibodies accompanied with higher levels of proinflammatory cytokines (34), augmented CD8+ T-cell induction (35), and the deficit in antibodies that bind to inhibitory Fc receptors on dendritic or CD8+ T cells (36, 37). In our cohort, an increased frequency of CD8^+^ effector memory T cells was indeed observed. Besides, it was reported that the extent of B-cell repopulation at the time of vaccination correlated well with subsequent antibody responses (35, 38), suggesting the reemergence of peripheral B cells as a potentially better marker than the time interval (14, 39). As shown in our study, the recovered immunogenicity was better captured by a cutoff of B-cell counts over 30 cells/μL rather than any specific time interval between vaccination and the last rituximab infusion. On the other hand, patients with adequate antibody responses were found to have significantly lower CD4^+^ central memory T-cell counts and higher percentages of exhausted CD4^+^ T cells, possibly because of skewed cytokines that are more in favor of plasma cell development (40–42).

The limitations of this study include a relatively small sample size and variability in post-rituximab vaccination timing. Additionally, although pemphigus patients included were all regularly followed up in our hospitals, and we would perform PCR tests if they become symptomatic or being in close contact with any COVID-19 patients, there is still a chance for the presence of asymptomatic infection. We also did not measure other parameters that could also correspond to vaccine protection such as nucleocapsid antibody titers.

In a holistic viewpoint, our study suggests that anti-CD20 treatments followed by a proper vacation until B-cell repopulation as a feasible strategy to determine the optimal vaccination window that preserves both humoral and cellular immunogenicity in patients with autoimmune disorders that could benefit from B-cell depletion therapy (43–45), and demonstrated the importance of an additional booster for these patients (46, 47). Considering the major role of cellular responses in anti-viral immunity (48, 49), azathioprine is not only recommended to be withheld in patients with active COVID-19 infection (50, 51), but also at the time of vaccination. Future studies with larger sample sizes are warranted to verify these findings.

## Data Availability

The raw data supporting the conclusions of this article will be made available by the authors, without undue reservation.
