# Supplementary figures and images for: Immunosuppressants in dermatology on vaccine immunogenicity: a prospective cohort study of pemphigus patients in the pandemic
Source: Front Immunol. 2024 Nov 22;15:1506962. doi: 10.3389/fimmu.2024.1506962 (PMC11621035; doi:10.3389/fimmu.2024.1506962)

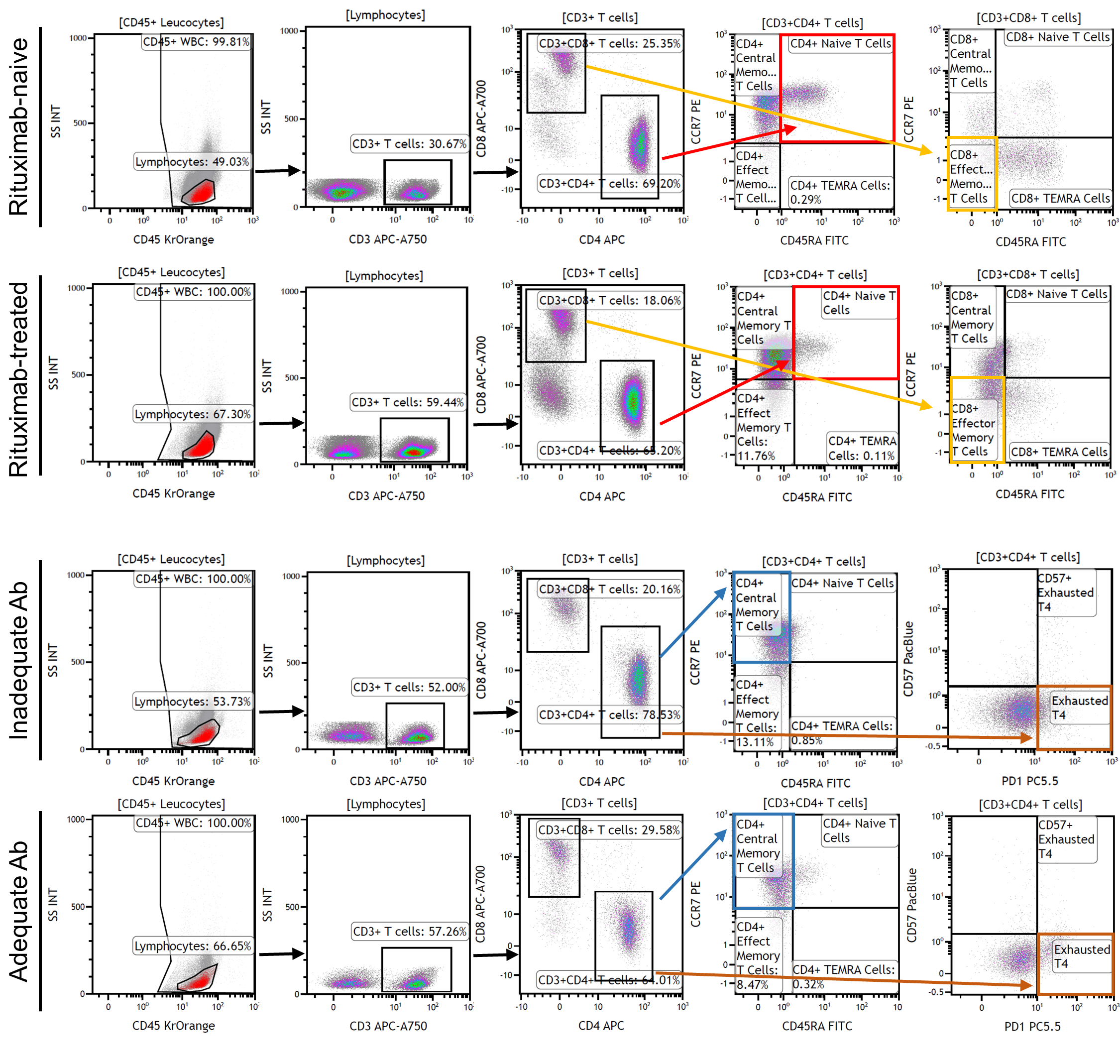

Supplement: Supplementary file 1 [file Image1.tif]

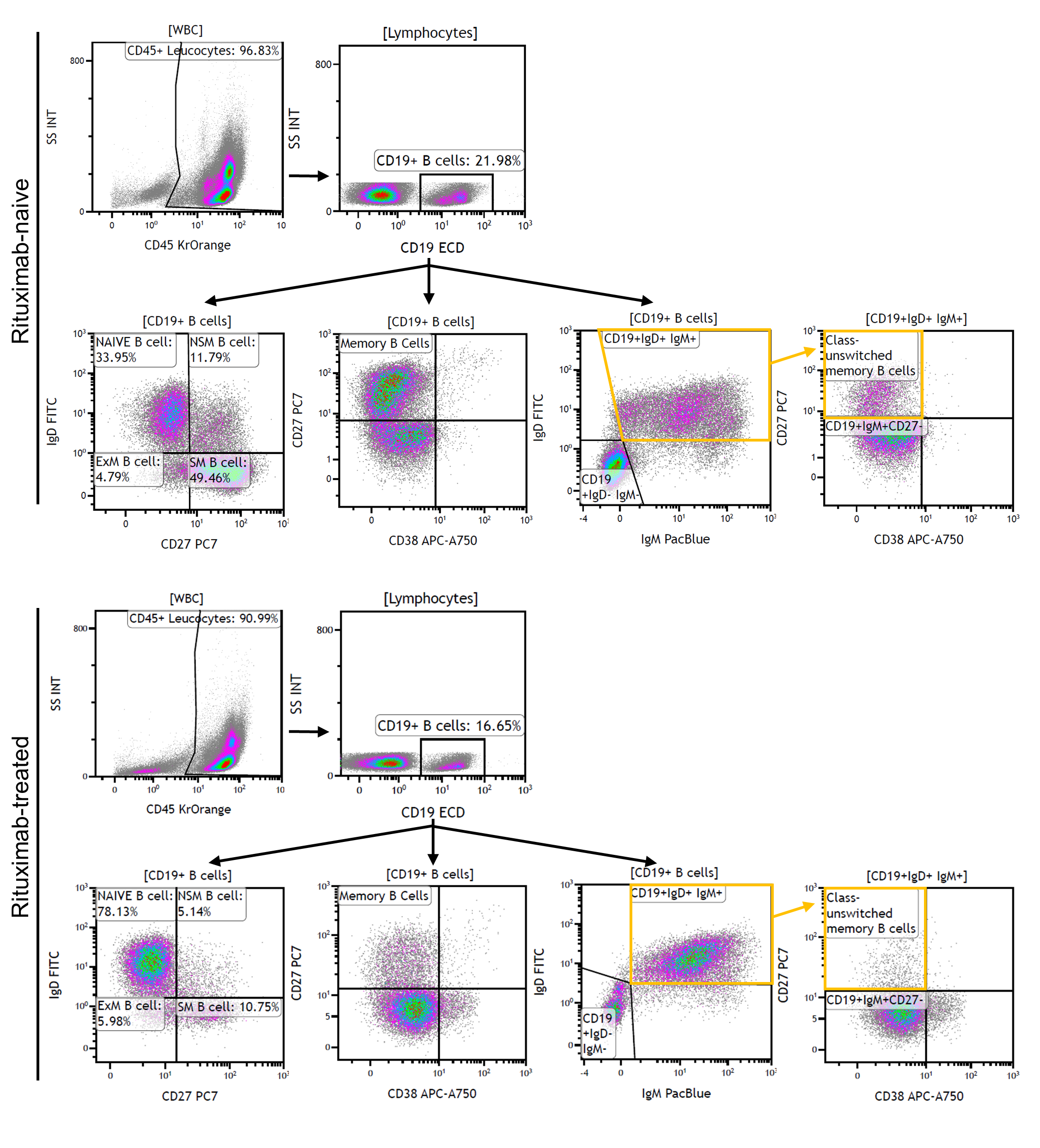

Supplement: Supplementary file 2 [file Image2.tif]
